# Pre-operative declining proportion of fractional anisotropy of trigeminal nerve is correlated with the outcome of micro-vascular decompression surgery

**DOI:** 10.1186/s12883-016-0620-5

**Published:** 2016-07-16

**Authors:** Fanfan Chen, Lei Chen, Wei Li, Ling Li, Xiangdong Xu, Weimin Li, Wuhua Le, Wei Xie, Hua He, Peng Li

**Affiliations:** Neurosurgery Department, Guangzhou First People’s Hospital Guangzhou Medical University, Guangzhou, 510180 People’s Republic of China; Neurosurgery Department, Shenzhen Second People’ s Hospital, The First Affiliated Hospital of Shenzhen University, Shenzhen, 518000 People’s Republic of China; Institutes of Brain Science, State Key Laboratory of Medical Neurobiology, Collaborative Innovation Center for Brain Science, Fudan University, Shanghai, 200032 People’s Republic of China; Recorder Department, Guangzhou First People’s Hospital, Guangzhou Medical University, Guangzhou, 510180 People’s Republic of China; Radiology Department, Guangzhou First People’s Hospital, Guangzhou Medical University, Guangzhou, 510180 People’s Republic of China; Neurosurgery Department, The Affiliated Hospital to Changchun University of Chinese Medicine, Changchun, 130021 People’s Republic of China; Department of Neurosurgery, Changzheng Hospital, Second Affiliated Hospital of Second Military Medical University, Shanghai, 200003 People’s Republic of China

**Keywords:** Trigeminal neuralgia, Type 2 trigeminal neuralgia, Magnetic resonance imaging, Neurovascular compression

## Abstract

**Background:**

Type 2 trigeminal neuralgia (TN) is an intractable neuropathic pain syndrome compared with type 1 TN because of the difficulty of diagnosis as well as the unsatisfactory prognosis. Neurovascular compression (NVC) is considered the major pathology of TN. Routine magnetic resonance imaging (MRI) sequences are inadequate for revealing the effect of NVC which is related to the surgical decision and outcome. The decreasing of fractional anisotropy (FA), one of the MRI diffusion tensor imaging (DTI) metrics, is correlated with the demyelination of trigeminal nerve (TGN) that reveal the severity of NVC.

**Methods:**

A retrospective review of patients treated with micro-vascular decompression (MVD) surgery was undertaken. All the patients were diagnosed as type 2 TN. FA of TGN of both sides were measured. The FA declining proportion = ((the mean FA value of healthy lateral)-(the mean FA value of the symptomatic lateral))/(the mean FA value of healthy lateral). Declining proportion of FA value, discovery of surgery and outcome of MVD were recorded and analyzed. Logistic regression analysis and linear regression analysis were employed to analyze the risk factors of declining proportion of FA value and MVD outcome.

**Results:**

Nineteen patients were assessed in our study. The average declining proportion of FA value for all patients was 0.25 ± 0.12. The average declining proportion of FA value of “success” and “failure” group was 0.32 ± 0.09 to 0.14 ± 0.10 (*P* = 0.002 < 0.05). The declining proportion of FA value of artery (including the artery plus vein situation) was 0.34 ± 0.06 in contrast to 0.15 ± 0.08 of vein (*P* = 0.000 < 0.05). MVD outcome was correlated with declining proportion of FA value (AUC = 0.900). Furthermore, declining proportion of FA value was higher in arterial compression situation.

**Conclusion:**

FA value quantitatively showed the alteration of TGN caused by NVC. It provided direct evidence about the effect of NVC which facilitated the diagnosis and surgical decision of type 2 TN. Besides, significant reduction of FA value may predict an optimistic outcome of MVD.

## Background

TN is characterized by sudden, usually unilateral, severe, brief, stabbing, recurrent attacks of pain in the distribution of one or more branches of the trigeminal nerve. It is believed that NVC at the root entry zone (REZ) is the main hypothesis based upon the high frequency of vascular conflict discovered during the operation and encouraging success of MVD surgery [1]. The effectiveness of pain relief post-surgery is 90 % [2] immediately and 58–78 % after five years [3].

During clinical course of TN treatment, a group of patients exhibit constant pain for more than 50 % of the time at the unilateral distribution of trigeminal nerve. This type of atypical pain is classified as an atypical TN or type 2 TN. As lacking typical symptom of pain attack, type 2 TN is more difficult to diagnose and make decision for MVD. Furthermore, the outcome of MVD for type 2 TN is less satisfied as for type 1 TN [4]. Whether a patient with atypical facial pain requires MVD and the prognosis is still a challenge. Time of flight (TOF) MRI sequence, a key preoperative examination is adopted extensively to clearly display the vessel/TGN contact in most cases [5, 6]. But whether the “contact” is responsible for pain onset requires more valuable information.

In recent years, the microstructural changes of trigeminal nerve have been discovered by new MRI sequences. The diffusion metrics of TGN revealed by DTI were utilized to display the pathological alterations of TGN. Liu et al. demonstrated that the value of FA was declined on the affected side compared with the unaffected side [7]. Moreover, it was reported that FA reduction correlated with the severity of clinic symptoms. Whereas whether FA value is significant to predict the surgical necessity and prognosis in type 2 TN is not reported by far.

## Methods

### Patient selection

This retrospective study was approved by ethics committee of Guangzhou First Municipal’s Hospital and Shenzhen Second People’s Hospital. From January 1, 2014 to September 30, 2015, totally 19 patients with type 2 TN were enrolled in Neurosurgery Department of Guangzhou First Municipal’s Hospital and Shenzhen Second People’ s Hospital for MVD. All patients were diagnosed as type 2 TN according to the following criteria: 1, idiopathic pain is located at the distribution of trigeminal nerve; 2, unilateral pain is described as aching, needling, throbbing, burning or other kind of sharp pain of moderate to severe degree; 3, constant pain that accounts for at least 50 % of the attacks [8]. All patients excluded other pathogenesis including cerebellopontine tumor or multiple sclerosis. None of the patients showed evidence of demyelinating disease on subsequent imaging or multiple sclerosis. All patients accepted the standard procedure of MVD. TGN were explored from REZ to the Meckel’s cave in all directions. The main demographic and clinical characteristics of the patients were listed in Table 1.

**Table 1:** Demographics of patients with type 2 TN

ID	Gender	Age	Side	Vessel	Declining proportion of FA value	Outcome
1	F	65	R	A	0.17	E
2	F	76	L	A	0.24	G
3	F	67	R	A	0.32	E
4	M	53	R	A	0.33	G
5	F	59	R	V	0.14	E
6	M	58	R	V	0.21	E
7	F	73	L	A	0.27	G
8	F	76	R	A	0.36	E
9	M	43	R	A	0.45	E
10	F	69	L	A	0.38	E
11	F	41	R	A	0.29	F
12	F	57	R	V	0.08	F
13	F	80	R	A	0.43	G
14	M	61	L	V	0.06	F
15	M	57	L	V	0.11	F
16	F	59	L	V	0.30	G
17	F	17	L	V	0.15	F
18	F	36	L	A	0.31	E
19	F	75	R	V	0.18	G

### MRI imaging

A sequence of 3D-TOF and DTI with other routine sequences were acquired (MAGNETOM Verio, SIEMEMS) in all 19 patients. The FA values of both sides of each patient were measured by three experienced radiologists that were blinded to the symptoms of the patients. Region of interest (ROI) was positioned at the TGN REZ or the sites near NVC. The mean FA values were acquired for both sides of each patient. The FA decreasing ratio was determined as follows: The FA declining proportion = ((the mean FA value of healthy lateral)-(the mean FA value of the symptomatic lateral))/(the mean FA value of healthy lateral).

### Follow up

All patients were follow-up at out-patient department or by telephone. Outcome was graded as “excellent” (E, free from facial pain), “good” (G, the facial pain was relieved or relapsed, but do not need medication or low-dose of medication and very little affection of daily activity) “failure” (F, no improvement or totally relapsed) at immediate post-operation and follow-up. As both excellent and good group were totally or largely free from the disturbance of TN, we integrated this two groups as a “success” group and compared with the “failure” group.

### Statistical analysis

Several statistic packages were utilized for data analysis including SPSS 13.0 (SPSS Corp, Chicago, IL, USA); Stata 12.0 (Stata Corp, College Station, TX, USA); and SAS 9.3,(SAS Institute, Cary, NC, USA). Means of declining proportion of FA value for different outcome or vessels were analyzed using Independent Sample *T* test (SPSS 13.0); Categorical comparisons were performed by Fisher exact test (Stata 12.0). Logistic regression analysis and linear regression analysis were employed to analyze the risk factors of MVD outcome and declining proportion of FA value, respectively (SAS 9.3).

## Results

### Patient characteristics

The average age of patients was 59.1 ± 15.9 years. None of the patients was bilateral TN. Eleven patients were right-sided and 8 patients were left sided. Female patients were 14, male were 5. The effectiveness of both groups was as follow: excellent outcome, 8 patients; good outcome, 6 patients; failure, 5 patients. The average declining proportion of FA value for all patients was 0.25 ± 0.12.

### MRI and surgical findings

**Fig. 1:**
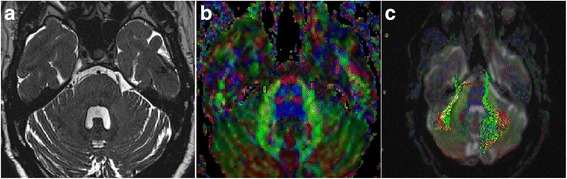
Pre-operative MRI of patient diagnosed as TN. **a** Routine MRI sequences displayed the nerve and vascular conflicting on the right side. **b** Diffusion tensor images and the region of interest measured of bilateral TGN. **c** The reconstruction of TGN fibers is shown

**Fig. 2:**
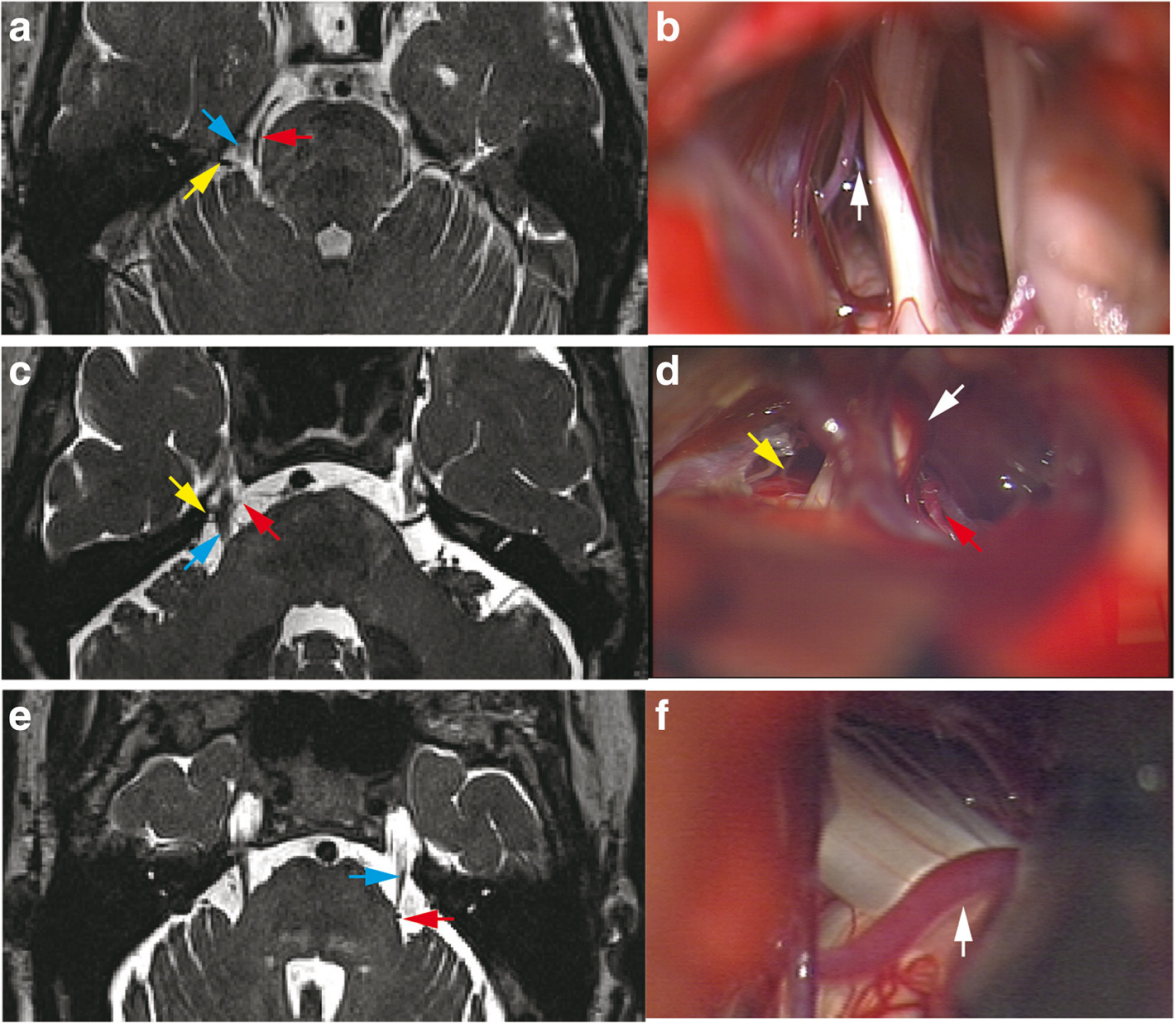
Routine MRI displaying NVC may have different intra-operative findings. **a** The superior cerebellar artery (SCA, red arrow) and a vessel (yellow arrow) located at the lateral of TGN (blue arrow) were the suspected vessels of NVC. **b** Intra-operative image showed SCA was close to but not in touch with TGN. A trivial vessel (vein) crawled on TGN. The white arrow points to the gap of the SCA and TGN. **c** MRI revealed the SCA (red arrow) and another vessel (yellow arrow) each formed a loop conflicting TGN (blue arrow) from inner side and outside. **d** Intra-operative image showed both vessels did not compress TGN (correspondingly marked with red and yellow arrow). A vein (white arrow) compressed the TGN from lower, outside at REZ to the TGN surface. **e** A very tiny vessel was displayed by pre-operative MRI at the outside of REZ of left TGN (blue arrow) which was easily neglected (red arrow). **f** An artery was found compressing the REZ of left TGN during surgery (white arrow)

Pre-operative MRI is important for surgical decision and instructive for surgical procedure (Fig. 1a). But the routine MRI sometimes offered limited information for the surgeons. As shown in Fig. 2a, b, the superior cerebellar artery (SCA) was the suspected vessel of NVC. The declining proportion of FA value of this case was 0.08. During operation we discovered that the SCA was close to but not in touch with the TGN. A trivial vessel (vein) crawled on the TGN which we doubted the influence of compression. We cautiously checked the overall length of TGN for not missing any possible responsible vessel but nothing found. We sufficiently loosened every part of TGN, and cautious coagulated the tiny vein to avoid TGN injury. The pain did not alleviate after MVD. A similar situation was observed in another patient. In Fig. 2c, d, MRI revealed the SCA and another vessel each formed a loop conflicting TGN from inner side and outside. The declining proportion of FA value was 0.21. But actually both vessels did not contact the TGN. A vein compressed the TGN from lower, outside at REZ to the TGN surface. We separated the contact at REZ and coagulated the vein (not the TGN), the patient got an excellent outcome. For Fig. 2e, f, a very tiny vessel was displayed at the outside of REZ of left TGN which is easily to be neglected. The declining proportion of FA value is 0.38. During MVD, an artery was found compressing the REZ of left TGN. The outcome of patient was excellent.

### The declining proportion of FA value was higher in patients with better MVD outcome or artery compression

**Fig. 3:**
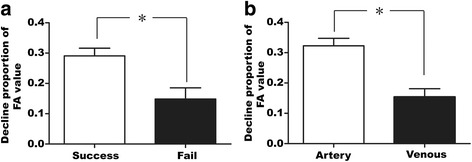
Declining proportion of FA value was statistically significance for different vessel or outcome. **a** The average declining proportion of FA value of “success” and “failure” group was 0.32 ± 0.09 to 0.14 ± 0.10 (*P* = 0.002 < 0.05). **b** The declining proportion of FA value of artery (including the artery plus vein situation) was 0.34 ± 0.06 in contrast to 0.15 ± 0.08 with vein (*P* = 0.000 < 0.05)

The overall complete relief of our patient with MVD was 42.1 % (8 of 19 patients). Fisher exact test was used to analyze gender and vessel for different MVD outcomes and displayed that none of them was statistically significant (*p* = 0.60 > 0.05 and *p* = 0.07 > 0.05, respectively). Independent-samples *T* test was employed to analyze the declining proportion of FA value for different MVD outcome. The average declining proportion of FA value of “success” and “failure” group was 0.32 ± 0.09 to 0.14 ± 0.10 (*P* = 0.002 < 0.05, Fig. 3a) which revealed that the declining proportion of FA value is higher of success group than failure group. The patients with higher declining proportion of FA value were more likely benefit from MVD.

Previous article reported that the artery is the main cause of TN [9]. Of our cases, artery accounted for 57.9 % cases (11 of 19 patients), including artery mixed with vein cases) and vein accounted for 42.1 % cases (8 of 19 patients). As we know, the vein vascular wall is thinner and the pressure is lower than artery it seems rational to deduce that the compression of vein is not as severe as artery. In our research the declining proportion of FA value of artery (including the artery plus vein situation) is 0.34 ± 0.06 in contrast to 0.15 ± 0.08 with vein (*P* = 0.000 < 0.05, Fig. 3b). It seems artery conflicts and demyelinates TGN more severely.

### High declining proportion of FA value was inclined to result in better MVD outcome

**Fig. 4:**
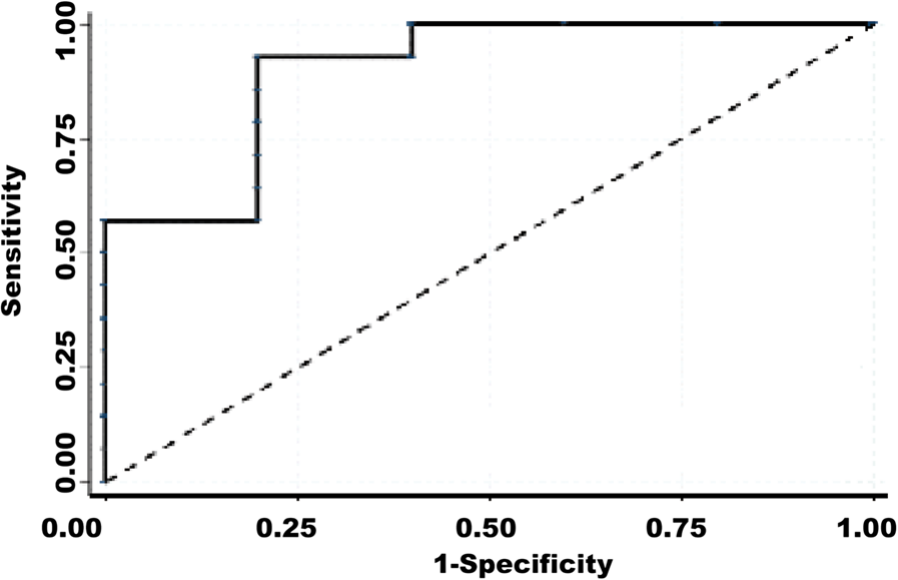
MVD outcome was correlated with Declining proportion of FA value. Discrimination accuracy for predicting outcome risk in major characteristics was assessed by the area under the curve (AUC) of receiver operating characteristic (ROC) curves. Here AUC = 0.900, (95 % CI: 0.7231-1.000) showed that MVD outcome was closely related to declining proportion of FA value. With the actual situation of data, it revealed that MVD outcome was inclined to high declining proportion of FA value

To determine the factors influencing outcome, logistic regression analysis was employed to assess the association between possible risk factors and outcome. In univariate analysis, the relation between the variables and the outcome were estimated. Odds ratio (OR) of gender, age, vessel and declining proportion of FA value were 0.41 (*p* = 0.42), 0.93 (*p* = 0.09), 10.00 (*p* = 0.07), 0.001 (*p* = 0.03), respectively. Then stepwise multivariate regression analysis was employed to reveal that declining proportion of FA value affected MVD outcome. Patients with high declining proportion of FA value had a significantly decreasing risk of “Failure” outcome (OR = 0.001, 95 % confidence interval (CI): 0.001-0.135; *P* = 0.033 < 0.05). In another word, high declining of FA value is more likely to benefit from MVD. Discrimination accuracy for predicting outcome risk in major characteristics was assessed by the area under the curve (AUC) of receiver operating characteristic (ROC) curves. Here AUC = 0.900, (95%CI: 0.7231-1.000) showing that MVD outcome is closely related to declining proportion of FA value. With the actual situation of data and clinical findings, it revealed that MVD outcome was inclined to high declining proportion of FA value (Fig. 4).

### Artery and “success” outcome is more likely to present high declining proportion of FA value

**Fig. 5:**
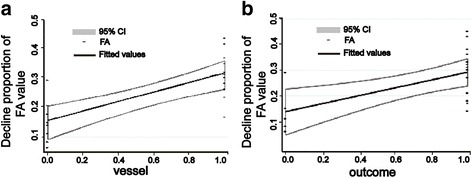
Different vessel and outcome displayed different declining proportion of FA value correspondingly. **a** Declining proportion of FA value was affected by vessel (*p* = 0.001 < 0.05, “0.0” for vein and “1.0” for artery.). Artery was inclined to present high declining of FA value. **b** MVD outcome of success presented with high declining proportion of FA value (*p* = 0.045 < 0.05, “0.0” for Failure and “1.0” for success.)

To determine the possible factors affected the declining proportion of FA value, linear regression analysis was used to assess the association between possible risk factors and declining proportion of FA value. After univariate analysis, we found vessel and outcome were potential risk factors. In the multivariable analysis, declining proportion of FA value was correlated with artery (*p* = 0.001 < 0.05, Fig. 5a) and “success” outcome (*p* = 0.045 < 0.05 Fig. 5b). These results just clarified that artery and “success” outcome was more likely to present high declining proportion of FA value.

## Discussion

According to Kajetan L. et al., up to 82 % of TN patients consulted their dentist first, and more than half of these patients received invasive dental treatments [9]. Although there is no data concerning the portion of type 2 TN patients who appealed to the dentist, the atypical symptom of type 2 TN is accompanied by persistent pain and usually with no definite triggering feature render it is more difficult to differentiate TN from other atypical facial pain including dental problems. Although there were some articles revealed that the effectiveness of MVD in type 2 was just as well as type 1 TN in defined conditions [10], the main conclusion to date was that the prognosis was less encouraging compared with type 1 TN [11–13]. This dilemma of diagnosis and treatment for type 2 TN requires further research in this field.

### NVC play an important role in type 2 TN

To better diagnose and treat type 2 TN, it is important to determine the mechanism that underlies type 2 TN. Although there were some researchers pointed out the character of pain was more important than the percentage of time of the pain [12], the most important nature of type 2 TN compared with type 1 TN is the constant feature which accounts for more than 50 % of the time. The mechanism for the differences of these two kinds of TN maybe that types 1 and type 2 TN represent the progression of TN spectrum. In a later stage of TN, paroxysmal lancinating pain progresses to a constant pain which may co-exist with the previous type 1 kind pain [14]. This alteration of pain character may results from structural or pathophysiological change within the pain pathways. A hypothesis of central sensitization mentioned that compression at the REZ of TGN by blood vessels resulted in demyelination of axons [11]. If not treated early, the damage of axons led to increasing central sensitive to stimulus including normal impulses. And this explained why type 2 TN was refractory to MVD: although the abnormal impulses resulted from vascular conflict is eliminated, the increased central sensitization led to the constant pain evoked by innocuous, slight sensation.

From the information above, though other etiology like central factor may participate in the pathogenesis of type 2 TN, Vascular compression or vascular contact (artery or vein) is still the cause or initial factor of the TN. The existing of NVC usually refers to MVD straightforward. While in a prospective research of the relation of NVC and clinical characteristics utilizing 3.0 Tesla MRI, Stine Maarbjerg et al. found that NVC was not associated with age or the natural history of TN (type 1 TN) [15]. Furthermore, the persistent pain of trigeminal nerve was not related to NVC either. In another word, type 2 TN was not associated with the NVC. This findings was supported by a recent research of Ko AL et al. which disclosed that NVC is not sufficient for TN [1]. The conclusions were based on the findings that NVC were both presented in TN and healthy people. And the TN patients may have NVC or not. Nevertheless, both authors still admitted that NVC played an important role in TN pathogenesis, not to mention most of the published articles still were certain about the NVC in TN [6, 16, 17].

### The differences of NVC

The discrepancy of the effect of NVC in TN requires more information about the NVC. Whether NVC certainly affects the microstructure of the trigeminal nerve in a specific patient is usually unknown. Indeed, according to Maarbjerg et al. the definition of NVC is defined as contact between a blood vessel and the trigeminal nerve without visible cerebrospinal fluid between the two structures. Furthermore the NVC may be “severe” or “simple” based upon the MRI imaging. Some studies have reported the compression of artery is more severe than that of vein indicating differences in NVC. However the determination of NVC severity mainly relied upon the experience of the doctor and few of the previous researchers accurately studied the effect of NVC following the pathological alteration of the trigeminal nerve structure. Upon the information of routine MRI, the position or relation of the trigeminal nerve with conflicting vessel maybe revealed, while the severity of the compression is subjective to some extent. An objective indicator displaying the extent of compression may contribute to surgical decision and the prognosis of MVD.

Actually, previous research about histopathology of nerve root specimens from patients with NVC exhibited dysmyelinating pathology [18], whereas samples from patients without NVC show only normal age-related alterations [19, 20]. Furthermore, MRI technique of diffusion-tensor imaging which can reveal valuable information of microstructure of the TGN [7], directly demonstrates the dysmyelination of TGN caused by NVC. It offered an opportunity to determine whether a NVC is pathological or harmless by noninvasive MRI techniques in contrast to routine MRI sequences.

### FA value helps to judge the severity and etiology of NVC

Improving NVC diagnosis in type 2 TN by MRI has received attention of many researchers [5–7]. High-resolution 3D MR imaging reconstruction was adopted in patients with constant facial pain (Type 2 TN) to help determine the presence/absence of neurovascular compression [5]. This MRI technique demonstrated the relation between vascular and TGN, but the alteration of TGN was not clear. It was reported demyelination without significant axonal injury was the essential pathological basis of the affected TGN [7]. DTI can quantitatively assess the microstructural abnormalities of the affected TGN in patients with TN by multiple diffusion metrics. The MRI DTI metrics FA representing the most valuable diffusion tensor imaging index that has been widely used to investigate white matter changes, decreased at the root entry zone of affected nerves [21, 22]. Furthermore, the decreasing of FA value was observed in many studies of the affected TGN and was more consistent than other DTI metrics, for example, the mean diffusivity (MD) [22, 23]. FA value quantitatively reveals the myelin structure change of trigeminal nerve and may be a potential objective MRI biomarker related with clinical severity [24]. All the knowledge mentioned above indicates the more decreasing of FA value, the more definite and serious of the vessel compression. This also means the prognosis of MVD is potentially more satisfactory. As we know, the features of vein include thinner vessel wall, relatively lower pressure and no pulsatile pressure compared with artery. These structural characteristics result in the compression of vein less severe than artery. This is in consistent with our findings that the declining proportion of FA value of artery compression was more significant than that of vein. These findings are especially valuable for type 2 TN as the clinical symptom is not typical and diagnosis is relatively difficult compared with type 1 TN. With the help of DTI sequences, a patient with constant facial pain excluded for other etiology (dental problem, intracranial tumor and so on) accompanied with the NVC and obvious decreasing of FA value may more reliably refer to MVD. Furthermore, the outcome of MVD may be more optimistic.

There are some limitations about this study. First of all, the sample size is relatively small and large scale study is required for further research. Secondly, the diagnosis of type 2 TN is mainly based on the symptom described by the patients. Selection bias may be unavoidable. The last, information of follow-up including MRI will contribute to the comprehensive understanding of the significance of FA value.

## Conclusion

TN is a more complicate disease than we thought previously due to satisfactory outcome of MVD in most situations. Type 2 TN is especially a challenge for neurosurgeons as the symptom is not typical and the outcome of MVD is less satisfactory. This study revealed that the declining proportion of FA value was related to severity, vessel type of NVC and prognosis concerning MVD. Combination of clinical symptom, the existing of NVC and FA value is helpful for diagnosis and MVD determination. We hope to further study the relation of NVC and FA value in a large cohort of patients in the future which may reveal the concrete value determining the effectiveness of NVC and MVD surgery.

## Abbreviations

AUC, Area under the curve; CI, Confidence interval; DTI, Diffusion tensor imaging; FA, Fractional anisotropy; MRI, Magnetic resonance imaging; MD, Mean diffusivity; MVD, Micro-vascular decompression; NVC, Neurovascular compression; ROC, Receiver operating characteristic; ROI, Region of interest; REZ, Root entry zone; SCA, Superior cerebellar artery; TGN, Trigeminal nerve; TN, Trigeminal neuralgia
